# Hierarchical Microtextures Embossed on PET from Laser-Patterned Stamps

**DOI:** 10.3390/ma14071756

**Published:** 2021-04-02

**Authors:** Felix Bouchard, Marcos Soldera, Robert Baumann, Andrés Fabián Lasagni

**Affiliations:** 1 Institut für Fertigungstechnik, Technische Universität Dresden, George-Baehr-Str. 3c, 01069 Dresden, Germany; marcos.soldera@mailbox.tu-dresden.de (M.S.); robert.baumann1@tu-dresden.de (R.B.); andres_fabian.lasagni@tu-dresden.de (A.F.L.); 2 PROBIEN-CONICET, Dto. de Electrotecnia, Universidad Nacional del Comahue, Buenos Aires 1400, Neuquén 8300, Argentina; 3 Fraunhofer-Institut für Werkstoff- und Strahltechnik (IWS), Winterbergstraße 28, 01277 Dresden, Germany

**Keywords:** direct laser writing, direct laser interference patterning, hot embossing, hierarchical structures, stainless steel, polymer, water contact angle, polyethylene terephthalate

## Abstract

Nowadays, the demand for surface functionalized plastics is constantly rising. To address this demand with an industry compatible solution, here a strategy is developed for producing hierarchical microstructures on polyethylene terephthalate (PET) by hot embossing using a stainless steel stamp. The master was structured using three laser-based processing steps. First, a nanosecond-Direct Laser Writing (DLW) system was used to pattern dimples with a depth of up to 8 µm. Next, the surface was smoothed by a remelting process with a high-speed laser scanning at low laser fluence. In the third step, Direct Laser Interference Patterning (DLIP) was utilized using four interfering sub-beams to texture a hole-like substructure with a spatial period of 3.1 µm and a depth up to 2 µm. The produced stamp was used to imprint PET foils under controlled temperature and pressure. Optical confocal microscopy and scanning electron microscopy imaging showed that the hierarchical textures could be accurately transferred to the polymer. Finally, the wettability of the single- and multi-scaled textured PET surfaces was characterized with a drop shape analyzer, revealing that the highest water contact angles were reached for the hierarchical patterns. Particularly, this angle was increased from 77° on the untreated PET up to 105° for a hierarchical structure processed with a DLW spot distance of 60 µm and with 10 pulses for the DLIP treatment.

## 1. Introduction

Nature provides countless surface structures that influence the macroscopic physical properties. Famous examples are the self-cleaning effect on the lotus leaf, the anti-bacterial behavior of spring-tail skin, and the high adhesion skin on gecko feet [1,2,3]. All these structures have in common that surface features with different size scales are combined, normally in the micrometer range with nanometer substructures. In the last decades, a lot of effort was spent to mimic these hierarchical structures and their outstanding properties in polymer surfaces, such as for example polyethylene terephthalate (PET). Due to its low weight, chemical stability, optical transparency, and low price, PET is widely used in many fields like packaging, food industry, textiles, electronics, and biomedical devices [3,4,5]. Especially in the food industry, pharmaceutical packaging, and biomedical applications, the non-wetting and easy-to-clean PET surfaces are of special interest due to the intrinsic antibacterial potential [6,7]. There are several innovative strategies to manipulate the wetting behavior of polymer surfaces, for instance plasma activation, thermal drawing, nanoparticles deposition, or applying functional coatings [8,9,10,11,12]. However, these methods are mainly devised for small areas and are not directly scalable to industrial throughputs. Moreover, these methods either require chemical treatments or induce a chemical modification of the treated surface, which might be detrimental for its compatibility with food, pharmaceutical or biotechnological products.

A convenient approach to replicate a microstructure on a polymer is hot embossing, whereby the texture of pre-structured master tools is transferred to a thermoplastic by applying heat and pressure. Due to the simple and low temperature processing, the final surfaces are free of toxic or chemically modified leftovers. According to the required processing throughput and final textured area, hot embossing can be implemented in plate-to-plate, roll-to-plate or roll-to-roll systems [13,14,15]. Therefore, this method can be tailored to specific application requirements with low costs and high productivity.

A key aspect for obtaining high-quality polymer replica is the morphology of the embossing tool, which is the inverse of the desired surface structure. Different processes have been employed to produce hierarchical microstructures on hard molds, such as soft lithography, etching followed by coating, electroplating, or electrical discharge machining [3,16,17,18,19,20]. Hierarchical surface geometries in the range of 100 nm to 800 µm already have been reported [17,18]. While these methods allow high-precision processing, they become more expensive and complex upon treating larger areas, especially for cylindrical tools needed in roll-to-roll hot embossing systems. An alternative solution for producing hierarchical textured molds is laser processing, a technology that already proved as a powerful tool for addressing the challenges concerning flexibility, versatility, and high throughput simultaneously [21,22,23,24]. The most common laser-based texturing technique is direct laser writing (DLW), which is used for processing a wide range of materials with a typical resolution between 10 µm up to 200 µm. Besides, DLW is a single-step and well-established technique for processing various structure sizes on metal surfaces [25,26,27]. When it comes to patterning of smaller features, on the order of a few µm, and with high throughput, direct laser interference patterning (DLIP) has become a reliable technique in recent years [28]. In DLIP, multiple laser beams are overlapped on a surface, leading to a periodic intensity distribution. The shape of the pattern can be controlled by the number of beams and their polarization. Overlapping two beams allows the generation of line-like features, whereas four beams produce dot-like patterns. In contrast to other laser processes, DLIP allows the period and depth of the generated structures to be easily adjusted by the angle of incidence of the beams and the accumulated fluence, respectively. For instance, micropatterns with periods between 0.5 µm to 20 µm and depths of up to ~5 µm have already been reported [28,29,30].

In this study, we show a strategy to create hierarchical patterns on PET foils inspired by the surface architecture of the lotus leaf. Stainless steel plates processed with DLW and DLIP served as masters, and their patterns were transferred to PET via plate-to-plate hot embossing. Finally, the wetting behavior of the hierarchical surface on the imprinted polymers was investigated.

## 2. Materials and Methods

### 2.1. Materials

Electropolished stainless steel plates (1.4301, Designblech GmbH, Erkelenz, Germany) with an initial roughness of S_q_ = 15 nm, dimensions of 80 mm × 60 mm and a thickness of 0.8 mm were laser-structured and subsequently used as masters for hot embossing.

Imprints were obtained on polyethylene terephthalate foils (PET, Pütz Folien GmbH, Taunusstein, Germany) with a thickness of 200 µm. PET is a thermoplastic with a melting temperature of 260 °C and a glass transition temperature of approximately 80 °C. Before imprinting, the foils were cleaned with a jet of compressed air and rinsed with pure ethanol.

To avoid sticking of the polymer during the hot embossing process, an anti-sticking layer was applied. Namely, the structured metal master was immersed in fluorophosphonic Acid C6 (Teflon)/isopropanol solution (molar concentration of 2 mmol/L) for one hour. Afterwards, the surface was rinsed with pure isopropanol and tempered at 150 °C for 10 min.

### 2.2. Laser Structuring Processes

**Figure 1:**
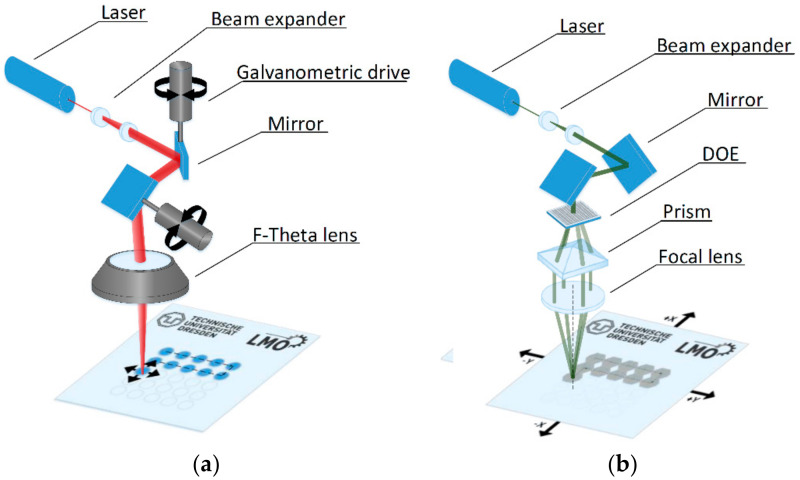
Schematics of (**a**) direct laser writing (DLW) and (**b**) direct laser interference patterning (DLIP). The DLW system operated at 1064 nm with pulse durations of 8 ns, 50 ns or 200 ns. For the DLIP technique, a laser beam was split with a diffractive optical element and recombined by a lens. The interference pattern was guided over the surface using 2 linear stages. Overlapping 4 beams lead to interference in the focal volume, resulting in an intensity with a dot-like distribution. Moving the target in X and Y directions with high precision linear stages, large areas can be structured.

Two laser-based manufacturing techniques were employed for texturing the metal master. First, direct laser writing (DLW) was used to pattern structures on stainless steel plates with a feature size in the range of 30 µm to 70 µm (GF machining solutions P 600, Biel, Switzerland). The experimental setup is illustrated in Figure 1a. This system consists of a nanosecond fiber laser emitting at a wavelength λ of 1064 nm and at a repetition rate of 30 kHz. The pulse width can be tuned from 4 ns to 200 ns. The laser beam was scanned on the material surface by using a high dynamic galvo-scanner system (Scanlab GmbH, Puchheim, Germany). The used f-theta lens with a focal distance of 254 mm yields a spot diameter in the focal plane of approximately 60 µm, resulting in a maximum scan field of 100 mm × 100 mm. By using an additional axis system, it is possible to treat large samples by stitching the single scan fields together.

After the DLW processing step, micropatterns were produced on the structured metal stamps using the direct laser interference patterning (DLIP) technique. The experimental setup is shown in Figure 1b. Here, a picosecond solid state laser source (Neolase GmbH, Hannover, Germany) emitting at a wavelength of 532 nm and a pulse width of 70 ps was used. In addition, the system can emit single pulses up to a repetition rate of 30 kHz, with a maximum average output power of 2.7 W (at 10 kHz). For generating the DLIP pattern, the initial laser beam is split into four sub-beams by a diffractive optical element. Then the sub-beams are parallelized by a prism and recombined by a focal lens on the material surface. At the maxima positions of the periodic interference pattern, the material is ablated producing a dot-like texture on the sample. The spatial period Λ of the dot-like patterns with four-beam interference can be calculated according to Equation (1) [31]:

(1) $$\Lambda =\frac{\lambda }{\sqrt{2}\cdot \sin\frac{\theta }{2}}$$

where θ is the interference angle and λ the wavelength of the utilized laser source. Within this study, a fixed spatial period of 3.1 µm was used. Additionally, the samples were mounted on an X-Y translation stage (Aerotech ABL-1500-E318187, Pittsburgh, PA, USA), enabling the possibility to adjust the pulse-to-pulse overlaps in both directions.

### 2.3. Plate-to-Plate Hot Embossing

**Figure 2:**
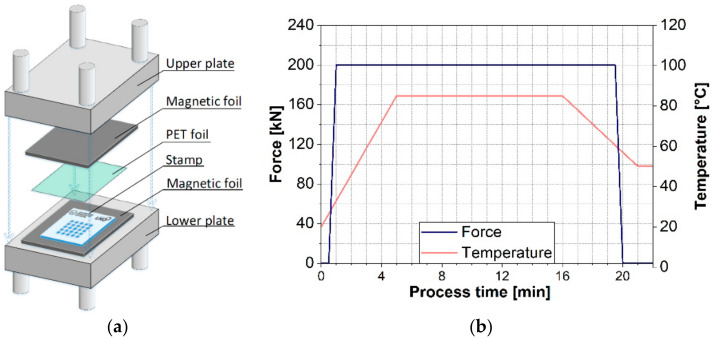
(**a**) Schematics of plate-to-plate hot embossing. (**b**) Applied forced and temperature as a function of process time for hot embossing experiments, performed within this study.

For plate-to-plate hot embossing, a temperature controlled hydraulic press (Paul-Otto Weber GmbH, Remshalden, Germany) was used. Prior to hot embossing, the structured metal master was cleaned with isopropanol in an ultrasonic bath for 30 min and coated with an anti-sticking layer, as described in Section 2.1. The experimental setup is schematically shown in Figure 2a. The PET foil was placed between the structured metal master and an untreated electropolished steel plate as counterpart. To homogenize the pressure distribution, a polyurethane-based magnetic foil with a thickness of approximately 1 mm was placed between each metal plate and the heating plates of the press.

After positioning the materials, the plates were pressed together, increasing the applied force from 0 to 200 kN within 30 s at room temperature. Considering the sample area of 4800 mm², a final pressure of 41.6 MPa was applied. Within the next 5 min, the temperature was raised to 85 °C, then it was held for 10 min and decreased to 50 °C in the following 5 min. All temperatures were set and controlled by a system integrated sensor with a variation of 2 °C for the given range. The pressure was released after reaching the final temperature within 0.5 min. After cooling down to room temperature, the PET foil was carefully removed by hand. In Figure 2b, the imprinting temperature and the applied force are shown as a function of the processing time. Due to low depths of the created structures, no additional investigations regarding the optimal process parameters were performed. The process parameters were chosen after preliminary experiments which are described elsewhere [32,33,34].

### 2.4. Surface Characterization

For investigating the surface topography of the micropatterned samples, an optical microscope (BX41M—LED Microscope, Olympus GmbH, Hamburg, Germany) with a 50× objective was used. Confocal microscopy (Sensofar S neox, Sensofar S.A., Barcelona, Spain) was employed at a magnification of 50× to measure the surface topography. The obtained topographical data were analyzed using the SensoMap software (SensoMap “Premium” Version 7, Sensofar S.A., Barcelona, Spain).

In order to identify and determine the structure heights corresponding to each single-scale texture within the hierarchical structure, the surface data were processed as follows: single profiles were extracted from topography data and filtered according to ISO 16610, using a robust Gaussian filter with a Cut-Off of 8.0 µm. This method is commonly used to determine the waviness and the roughness of a surface [35]. In this study, the method allowed us to measure the depth of the large-scale DLW structure as well as of the small-scale DLIP structures.

For a detailed evaluation of the structure on the metal master and the polymer foil, high-resolution surface images were taken with a scanning electron microscope (Gemini 982, Carl Zeiss AG, Oberkochen, Germany) at an acceleration voltage of 2 kV. The polymer samples were coated by sputtering with a 20 nm thick gold layer.

The static water contact angle (WCA) measurements were performed using a contact angle system (Krüss DSA 100 S, Hamburg, Germany) and a droplet volume of 4 µL of deionized water at ambient conditions, i.e., room temperature of 20 °C and relative humidity of 17%. The tangent droplet fitting method was used for all measurements to determine the WCA, and each measurement was repeated at least 5 times for a better statistical significance.

## 3. Results and Discussion

In the following sections, the three fabrication steps, namely direct laser writing (i), direct laser interference patterning (ii), and plate-to-plate hot embossing (iii) will be described in detail. For the laser processes, the influence of the process parameters on the topography of the metal surface was investigated. Due to the fact that the final imprint is the inverse structure of the hierarchical metal plate, the field of interest was set on evaluating the depth and the shape of the produced structures.

### 3.1. Direct Laser Writing Process

Arrays of micro-holes were produced with direct laser writing on stainless steel 1.4301. Fixed values for the laser fluence of 0.44 J/cm² and a repetition rate of 30 kHz were used. The distance between the pulses, the number of applied pulses, and the pulse duration were varied, resulting in different surface geometries.

**Figure 3:**
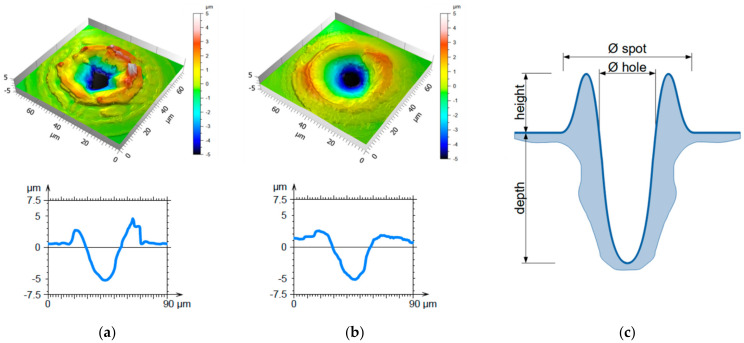
Confocal images and profiles of spots processed with DLW using (**a**) 70 pulses with a pulse duration of 8 ns and (**b**) 400 pulses at 200 ns. The laser fluence was kept constant at of 0.44 J/cm². The resulting holes had a depth of approximately 5.0 µm in both cases. (**c**) Schematical drawing of the cross section of a laser processed spot defining the structure depth, height of molten material, and spot and hole diameters.

Confocal microscopy was used to analyze the surface topography. In all processed samples, it was observed that the laser-patterned holes were surrounded by molten ring formations raising above the untreated surface, as shown in Figure 3a,b. These samples were processed with 70 (Figure 3a) and 400 (Figure 3b) pulses with pulse durations of 8 ns (Figure 3a) and 200 ns (Figure 3b), resulting in a crater depth of 5 µm in both cases. The confocal analysis revealed that the shorter pulses (8 ns, see Figure 3a), led to an inhomogeneous distribution of molten material surrounding the crater and a less rounded hole shape compared with the textures with longer pulses (cf. Figure 3b with a pulse duration of 200 ns). The influence of the pulse duration on the resulting topography can be explained by the corresponding variations in the thermal diffusion length, which represents the ability of the material to dissipate heat. It is well-known that shorter pulse durations lead to a shorter diffusion length and, therefore, a higher temperature on the area corresponding to the laser focus. Thereby, the temperature gradient between the center of the laser focus and the adjacent material is relatively large, resulting in a gradient of the surface tension of the molten material that drives a high amount of material to flow out of the melt pool that stacks around the spot. This process is commonly known as Marangoni convection [36,37,38]. In addition, for shorter pulse durations, the high temperatures present in the center of the spot increase the probability of spontaneous transitions from the liquid phase to the gas phase. Such transitions increase the pressure in the melting pool (recoil pressure), sputtering the molten material out of the laser spot center. Both the structure depth and the surrounding ring height can be determined from confocal microscopy images according to the definitions in the schematic shown in Figure 3c.

**Figure 4:**
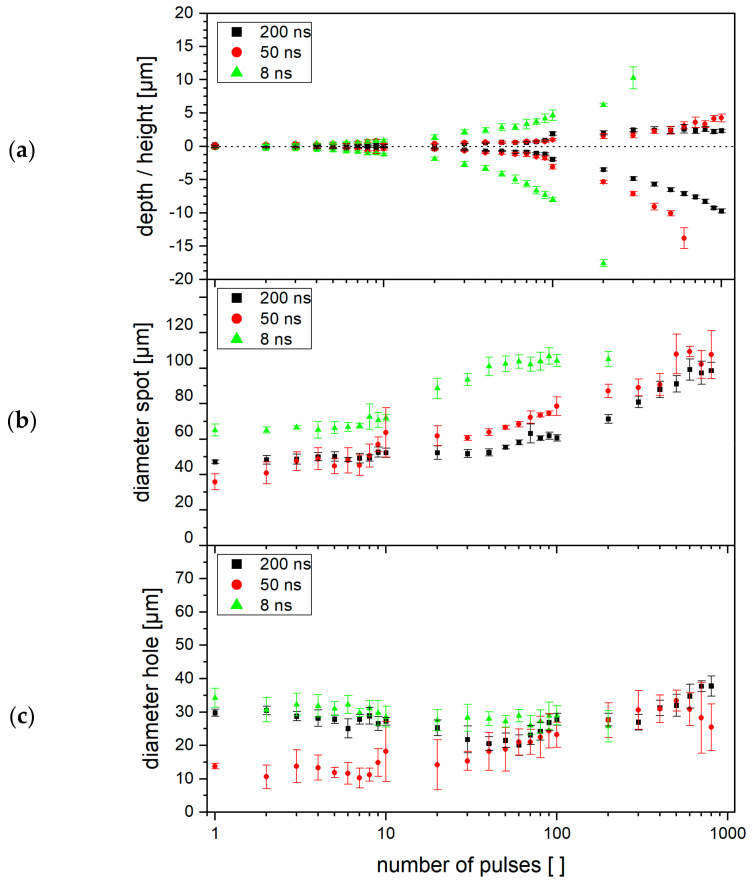
(**a**) Measured structure depth and height relative to the untreated surface, (**b**) spot diameter and (**c**) hole diameter for DLW processed holes on stainless steel according to the definition in Figure 3c. The error bars represent the standard deviation of 8 measured spots.

As can be seen in Figure 4a, the ring height as well as the depth of the craters decrease with increasing pulse durations (from 8 to 200 ns), in accordance with the Marangoni phenomenon described above. In turn, applying a higher number of pulses, more material was molten, stacking up around the hole and forming higher rings. Additionally, the diameters of the holes and spots were measured according to the schematics presented in Figure 3c. The results are presented in Figure 4b,c, for the three pulse durations used (5, 80, and 200 ns). As expected, higher numbers of applied pulses lead to larger hole and spot diameters for all pulse durations. In particular, for a pulse duration of 8 ns, the diameter tends to saturate at approximately 110 µm.

As shown in Figure 3, the surrounding area of the holes was covered by randomly distributed recast material. To remove these inhomogeneous bulges and smoothen the surface, a second DLW step was optimized to laser-polish the sample surface [39,40]. The DLW treated surface was scanned with a line-like laser path and with varying fluence from 0.42 µJ/cm² to 4.2 µJ/cm². Based on preliminary studies, the pulse duration was set to 200 ns, the scan speed to 7500 mm/s and the frequency to 500 kHz. This led to a pulse-to-pulse overlap of 15 µm in X- and Y-directions.

**Figure 5:**
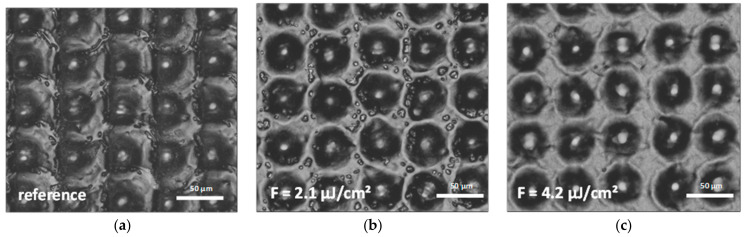
(**a**) Optical microscopy images of a stainless steel surface patterned with DLW with a spot distance of 60 µm using 500 laser pulses at a pulse duration of 200 ns and a fluence of 0.44 J/cm². (**b**) Patterned surface with an additional laser polish step at a fluence of 2.1 µ/cm² and (**c**) 4.2 µJ/cm².

Figure 5a shows an optical microscope image of a DLW-treated steel plate (processed with 500 pulses with a pulse duration of 200 ns and pulse-to-pulse distance of 50 µm), where recast material and debris around the holes can be observed. The laser polishing process permitted to eliminate the random recast material as well as the attached particles as shown in Figure 5b,c. It was also found that increasing the laser fluence from 2.1 µJ/cm² (Figure 5b) to 4.2 µJ/cm² (Figure 5c) led to an even more homogenous and flat surface between the holes. Confocal measurements revealed that the initial mean roughness of S_q_ = 2.5 µm of the DLW reference surface could be reduced to a minimum value of S_q_ = 1.9 µm, using a laser fluence of 4.2 µJ/cm² for the laser-polishing step.

Next, the influence of the pulse-to-pulse distance on the surface structure was investigated. For this purpose, hole-like arrays with different spot distances were processed. Based on the previous results, the samples were irradiated with 500 pulses at a pulse duration of 200 ns and a laser fluence of 4.2 J/cm². Additionally, the structured surfaces were laser polished, using also a pulse duration of 200 ns, a scan speed of 7500 mm/s, and a frequency of 500 kHz.

**Figure 6:**
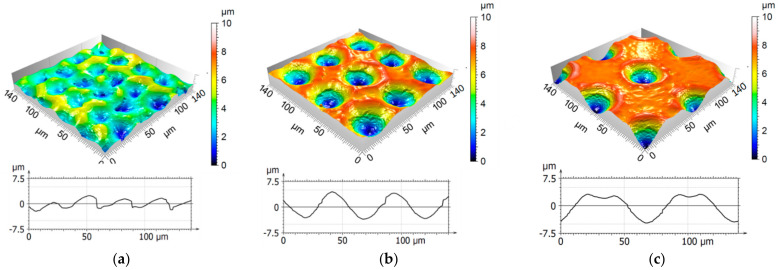
Confocal images and corresponding profiles of stainless steel surfaces micropatterned with 500 pulses with a pulse duration of 200 ns and a fluence of 0.44 J/cm². The DLW spot distance was set to (**a**) 30 µm, (**b**) 50 µm, and (**c**) 70 µm.

The surface topographies for the processed samples with a spot distance of 30 µm, 50 µm, and 70 µm and the corresponding profiles are illustrated in Figure 6. The sample patterned with a spot distance of 30 µm (Figure 6a) had a very shallow average depth of 2.9 µm and a very inhomogeneous topography. As the spot distance is smaller than the spot diameter, the melt pool flowed into the previously patterned holes deteriorating their quality and producing instead quasi-random structures. In contrast, a spot distance of 50 µm led to a homogenous periodic pattern with a uniform structure depth of 6.4 µm, as shown in Figure 6b. The recast material can be easily observed around the edge of every single DLW structure. Likewise, the surface structured with a spot distance of 70 µm (Figure 6c) resulted in a homogenous pattern with a structure depth of 7.2 µm.

### 3.2. Direct Laser Interference Patterning

For producing textured surfaces with higher resolutions, the DLIP technique was utilized. By overlapping four laser beams on the surface, a dot-like intensity distribution is obtained on the surface. Following Equation (1), the interfering angle was set to 14.4°, resulting in a spatial period of 3.1 µm for the used laser wavelength of 532 nm. The fluence was set to 0.17 mJ/cm² at a frequency of 10 kHz for all the experiments. The number of applied pulses was adjusted to 1, 5, and 10. The spot distance was set to 40 µm in X direction and 60 µm in Y direction.

**Figure 7:**
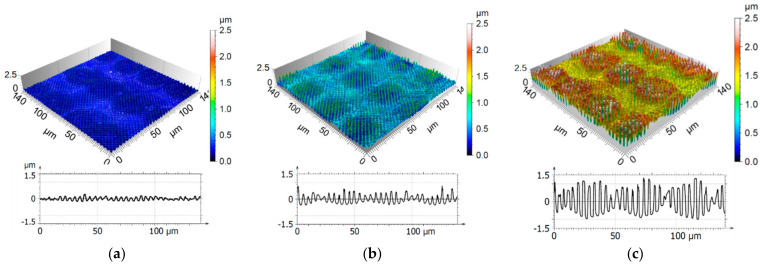
Confocal microscopy images of periodic microstructures and extracted profiles of DLIP structured stainless steel surfaces with (**a**) 1, (**b**) 5, and (**c**) 10 applied pulses. The measured average structure depth was (**a**) 0.23 µm, (**b**) 0.74 µm, and (**c**) 1.74 µm.

The confocal images, shown in Figure 7a–c, revealed the produced hole-like structure with a spatial period of 3.1 µm. While the number of applied pulses increased from 1 to 10, the average structure depth increased from 0.2 µm to 1.7 µm. For all number of pulses, the largest peak/valley ratio can be seen in the center of the spot. This effect can be explained by the fact that the laser source emits a beam with a gaussian energy distribution whose fluence maximum lies in the center. In addition, the molten material reached a higher level than the initial surface, as can be clearly seen in Figure 7c. This effect has been already observed in previous studies [41,42].

### 3.3. Plate-to-Plate Hot Embossing

The stainless steel master plates patterned with either single-DLW, single-DLIP, as well as the combination of both techniques were manufactured based on the previous results (Section 3.1 and Section 3.2). To remove particles and leftovers from the DLW process and to prepare the DLW-treated surface for additional DLIP-laser treatment, the DLW-treated samples were laser polished using a laser fluence of 4.2 µJ/cm² according to the results shown in Section 3.1.

For DLW, the selected process parameters were 500 pulses at a fluence of 0.44 J/cm² with varying spot distance from 30 µm to 70 µm in steps of 10 µm followed by a laser-polished step with 200 ns pulses at a fluence of 4.2 µJ/cm^2^. For the second level structure, the DLIP selected parameters were 1, 5, and 10 pulses and a laser fluence of 0.17 mJ/cm². Each structured field had a size of 10 mm × 10 mm. The laser structured metal master plate was later implemented in a plate-to-plate hot embossing system, for transferring the microtextures to the PET foils.

**Figure 8:**
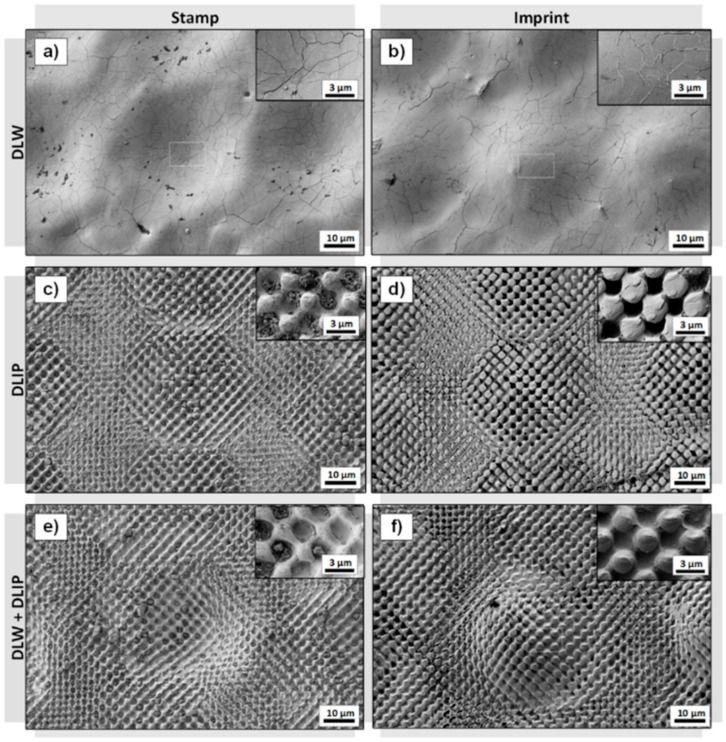
Scanning electron images of laser processed surfaces on stainless steel using (**a**) DLW technique, (**c**) DLIP technique, and (**e**) combined DLW and DLIP techniques. Surface structures were transferred to PET foil (**b**,**d**,**f**) using plate-to-plate-hot embossing. DLW structures were processed, using a 1064 nm pulse laser system, operating at 30 kHz and a pulse length of 200 ns. Spot distance was set to 70 µm. For DLIP, 10 pulses with constant fluence of 0.17 mJ/cm² were applied.

Figure 8 shows exemplarily SEM images of: (Figure 8a) a DLW-patterned sample with a spot distance of 70 µm, (Figure 8c) a DLIP-treated surface with 10 pulses, (Figure 8e) a hierarchical surface combining the single-scaled DLW and DLIP textures, together with the corresponding imprints on PET (Figure 8b,d,f). As it can be observed, the different single- and multi-scaled structures produced in the metal master were successfully imprinted on the polymer foil.

For the DLW structure, even sub-micrometer heat cracks with a width between 50 and 150 nm could be reproduced to the polymer, denoted by the narrow-walled elevations with a height of up to ~50 nm, as can be seen in Figure 8b (the heights were recorded using confocal microscopy). Heat cracks are typically caused by the high temperatures and fast cooling down during the nanosecond DLW process [43]. The overheated melt cooled down rapidly, leading to surface tensions, which were released as cracks on the surface.

In the case of the DLIP microstructures produced on the metal plate (Figure 8c), the holes reached diameters of ~2 µm. In comparison with the results of the DLW process, no cracks were observed on the DLIP treated sample. This might be attributed to the fact that the DLIP process, conducted with picosecond laser pulses, promoted material vaporization rather than melt flow. As mentioned before, the hot embossing process permitted to fabricate the inverse structure of the mold with a very high accuracy on the PET foil, as shown in Figure 8d.

**Figure 9:**
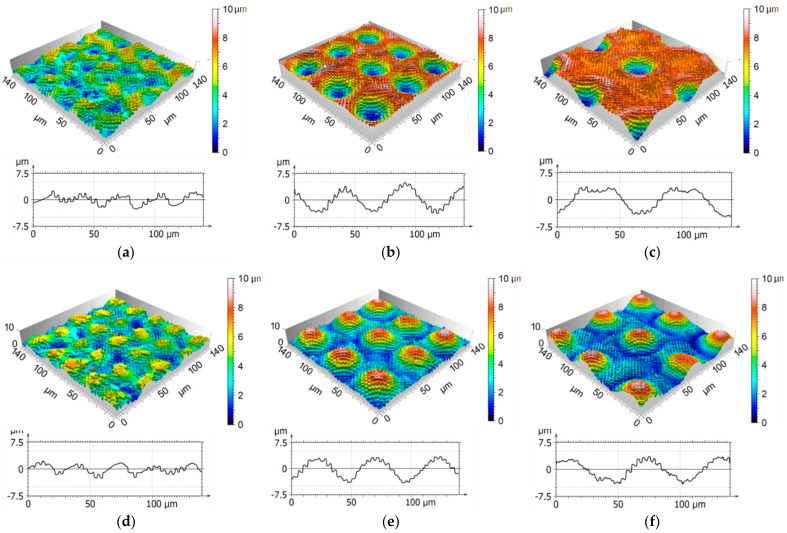
Confocal images and extracted profiles of hierarchical microstructures on stainless steel (**a**–**c**) and a hot embossed PET foil (**d**–**f**). Distance between spots was (**a**,**d**) 30 µm, (**b**,**e**) 50 µm, and (**c**,**f**) 70 µm. Profiles were extracted with a length of 140 µm at a range of 15 µm.

In addition, the hierarchical geometry of the metallic stamp produced by DLW and DLIP manufacturing techniques could be successfully imprinted on the polymer, as depicted in Figure 8f. Surprisingly, the SEM images of both stamp and imprint show no evidence of cracks. This effect can be explained by the ablative characteristic of the DLIP treatment as well as the very low depth of the cracks (~50 nm).Next, the topographies of the stamps and imprints measured by confocal microscopy are compared. Figure 9a–c shows as an example the topography of the hierarchical surfaces on the metal stamp, whereas the corresponding imprinted textures on the PET foil are presented in Figure 9d–f. The spot distances for the DLW process were 30 µm (Figure 9a), 50 µm (Figure 9b), and 70 µm (Figure 9c), and the DLIP fabrication step was performed using 10 pulses per position in this case. For all spot distances, the DLIP structure is perfectly visible reaching heights up to 1.7 µm (note that the spatial period of the dot-like pattern was 3.1 µm). For the smallest DLW spot distance of 30 µm (Figure 9a,d), the master as well as the imprint showed an inhomogeneous surface structure. During the laser process on the metal master, molten material refilled neighbored structures leading to a reduced depth. For the spot distance of 50 µm (Figure 9b), the material formed homogeneous walls around each spot and for a distance of 70 µm (Figure 9c), ring-like substructures with a height of up to 2 µm were observed.

The measurements also showed that the depth of the structures on the metal stamp were very similar to the heights of the structures on the imprints, suggesting that the softened polymer could successfully fill the cavities of the stamp during the hot embossing step. For example, the total structure depth on the master for a spot distance of 70 µm was 7.1 µm, whereas for the corresponding imprint the depth was 7.0 µm. The difference between stamp and imprint structure heights lies within the range of the measurement error, which is approximately 0.7 µm.

### 3.4. Wettability Characterization

Water contact angle (WCA) measurements have been performed on 10 mm × 10 mm sized square fields of imprinted PET samples to study the possibility of controlling the wettability characteristics with the produced microstructures. In each field, five droplets of deionized water with a constant size of 4 µL and diameter of 2 mm were positioned in the corners as well as in the center of each field. The contact angle was measured with the tangent drop profile fitting method [44].

**Figure 10:**
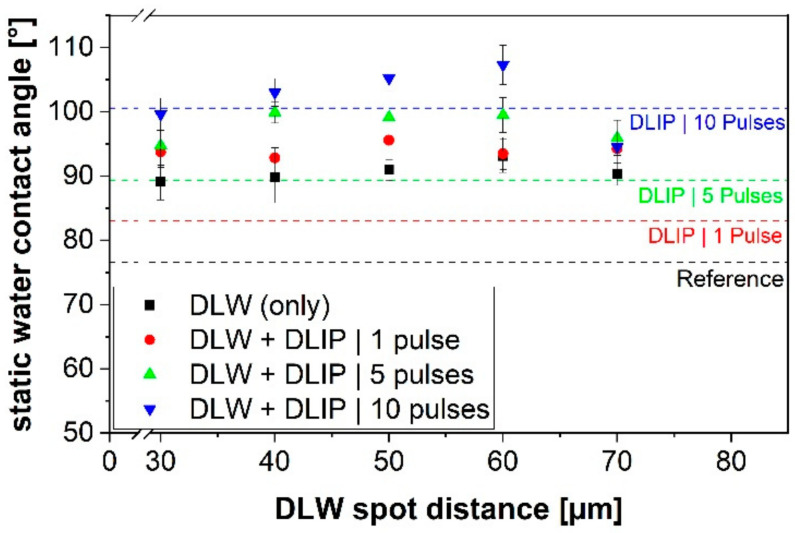
Measured static water contact angle on PET foils imprinted with different single- and multi-scale structures. Multi-scale structures were processed combining the DLW and DLIP techniques. Single-scaled DLW structures are shown as black values, whereas single-scaled DLIP structures are represented with dashed lines. Error bars indicates the mean value of 5 measurements. Black dashed line represents the reference on a flat PET surface.

The wettability characterization results for single- and multi-scaled structures are shown in Figure 10 as a function of spot distance for the DLW process and the number of applied pulses in case of the DLIP treatment. Unstructured PET is slightly hydrophilic characterized by a WCA of 76.7° ± 0.5° (black dashed line in Figure 10). In turn, all the structured PET surfaces reached a hydrophobic state. It was also observed an increasing trend of the WCA as the DLW spot distance increased from 30 µm to 60 µm, where the WCA reached the maximum value of 105° ± 2.0° on the hierarchical sample structured with 10 pulses for the DLIP treatment. Likewise, increasing the number of DLIP pulses led to an increase in the WCA for all the studied samples. These results show that the hierarchical patterns presented higher WCA than the corresponding single-scale textures based on either DLW or DLIP processes (WCA corresponding to the DLIP structures are shown in Figure 10 with dashed lines, red: 1 pulse, green: 5 pulses, blue: 10 pulses). The observed increase in the WCA can be related to an increase of the surface roughness as determined from confocal microscopy measurements. This behavior is a typical characteristic of the Cassie–Baxter model, that predicts an increase in the WCA as the surface roughness increases, independently of the initial WCA of the untreated surface [45]. The underlying principle of this model is that the water droplets sit on the top of the bumps of the texture resulting in confined air pockets between the valleys of the texture and the water droplet. As the roughness increases, the amount of trapped air increases, the contact area between solid surface and water droplet decreases, resulting in an increased WCA [46]. However, it has to be mentioned that the superhydrophobic condition, characterized by a WCA higher than 150°, could not be reached.

An interesting characteristic of the WCA measurements is the decrease of the contact angle for a spot distance of 70 µm in the DLW process. This effect was observed for the case of the DLW-treated sample as well as in all the hierarchical surfaces. This effect might be attributed to a less compact arrangement of the DLW holes, that leaves areas between the DLW features and thus increasing the droplet’s sitting area.

## 4. Conclusions

In this study, we presented a strategy to produce hierarchical surface structures on polyethylene terephthalate (PET) foils. To that end, a stainless steel master plate was micro-structured by combining two direct laser writing (DLW) and direct laser interference patterning (DLIP). Using DLW, holes with a diameter between 50 µm and 80 µm and depths up to 7.2 µm were produced. The quality of the surface of the DLW-treated sample was enhanced by a laser polish step, reducing the roughness from 2.5 µm down to 1.9 µm. Employing the DLIP method, dot-like geometries were produced, featuring a spatial period of 3.1 µm and depths up to 1.7 µm. Comparing confocal microscopy images and SEM images of the stamps and imprints, it was observed that the geometrical characteristics of the stamps (e.g., height of the DLIP features, depth of the DLW holes) could be very accurately transferred to the PET polymer foils. The wettability characterization revealed that the measured static water contact angle (WCA) on structured PET foils were in all cases higher than in the flat reference foil. The highest WCA were reached for the hierarchical patterns. In particular, this angle was increased from 77° on the untreated reference up to 105° for a hierarchical structure processed with a spot distance of 60 µm in the DLW step and with 10 pulses for the DLIP treatment. However, it is worth mentioning that the superhydrophobic behavior could not be reached and further investigations are needed, e.g., utilizing different geometries.

In this study, we demonstrated the feasibility of combining DLW, DLIP, and hot embossing to functionalize PET foils by imprinting hierarchical microtextures. However, only small sized areas (1 cm^2^) were tested in this work due to the relatively low processing speeds of the used laser-systems and the hot embossing step. To make this approach compatible to industrial processing, further technical improvements need to be implemented. For instance, the DLW and DLIP optics should be combined in the same laser system to avoid sample handling and alignment. In addition, the plate-to-plate hot embossing step should be replaced by a roll-to-roll process where polymer foils can be patterned at web speeds of tens of m^2^/min [34].

Finally, the long-term stability and robustness of the imprinted structures as well as their increased WCA could be an issue and will be investigated in detail in future works.

## Data Availability

The data presented in this study are available on request from the corresponding author.
